# Editorial: Reimagining roles and identity in the era of human - AI collaboration

**DOI:** 10.3389/fpsyg.2025.1736730

**Published:** 2025-11-26

**Authors:** Xi Chen, Ivan Wen, Qixing Qu, Wenjing Chen

**Affiliations:** 1 Management Science Department, School of Business and Tourism Management, Yunnan University, Kunming, China; 2 School of Travel Industry Management, Shidler College of Business, University of Hawai'i at Manoa, Honolulu, HI, United States; 3 School of International Trade and Economics, University of International Business and Economics, Beijing, China; 4 School of Economics and Management, Beijing University of Posts and Telecommunications, Beijing, China

**Keywords:** roles, identity, human–AI collaboration, artificial intelligence, AI for social good

Human civilization is entering an epoch of profound human–AI collaboration—an era in which interaction between humans and intelligent systems no longer belongs to speculation but defines a new frontier of interdisciplinary inquiry (Vaccaro et al., 2024). Within this emerging symbiosis, the essence of subjectivity and identity demands renewed scrutiny. The boundaries of the human—its agency, autonomy, and existential significance—are being redrawn in collaborative terrains where technology no longer serves merely as an instrument but participates intimately in cognition, perception, and decision-making (Fügener et al., 2022; Reinhardt et al., 2025).

Indeed, human–AI collaboration reshapes the very fabric of intersubjectivity. Artificial intelligence has evolved beyond a mechanical tool into a quasi-subjective partner in reasoning and creation (Hou et al., 2025). In this process, it unsettles established hierarchies of power, redistributes responsibility, and reconfigures the mechanisms of value co-creation (Wessel et al., 2025). Such transformation calls for reaffirming the distinctiveness of human cognition, emotion, and moral judgment—those fragile yet irreplaceable capacities that lend ethical texture to progress (Glickman and Sharot, 2025). At the same time, AI dissolves the once-stable boundary between reality and virtuality, transforming both the spaces and meanings of identity expression (Heinrich and Gerhart, 2025). Subjectivity now extends beyond the corporeal self into plural performances across digital dimensions—liberating yet perilous, emancipatory yet disciplinary. Hence, technological advancement must be tempered by humanistic care, preserving dignity within empowerment and conscience within innovation.

At its conceptual core, this rethinking of subjectivity invites deeper reflection on the nature of humanity and intelligence in the technological age. It is a dialogue that transcends disciplinary boundaries, drawing from sociology, psychology, management, communication studies, and computer science. Together, these fields seek to understand the co-evolution of human consciousness and artificial cognition. Three themes define this frontier: the psychological and interactive dynamics of human–AI collaboration; the repositioning of human uniqueness within intelligent ecosystems; and the ethical principles guiding digital identities in AI-mediated environments. In the end, these dimensions form the foundation for reimagining human subjectivity amid technological symbiosis.

Empirical research illuminates this landscape, revealing how personality, emotion, and resource dynamics shape human–AI relations. Liu and Chen find that Generation Z's chatbot-assisted purchases are shaped by extraversion, agreeableness, and openness, along with chatbot expertise and customization—underscoring the need for designs attuned to human individuality rather than uniform assumptions. Yu and Chang show that students' digital photograph hoarding arises from emotional attachment and fear of missing out, as AI tools increasingly serve as repositories of affect and memory. Han and Ren reveal that unequal access to AI can paradoxically enhance team productivity through complementary interaction, challenging the notion that equality in technology always yields optimal collaboration. Collectively, these studies expose the complex interplay of personality alignment, emotional mediation, and strategic asymmetry that transcends traditional human–human frameworks.

Beyond interpersonal dynamics, AI is also redrawing the contours of roles and agency in academic and professional life. Huang and Zhao demonstrate that AI literacy enhances wellbeing by fulfilling needs for autonomy, competence, and relatedness, thereby improving work–life balance and job satisfaction. Zhao and Huang extend this view, showing that AI literacy stimulates pedagogical innovation through strengthened attitudes, norms, and perceived control, moderated by resources and autonomy. Jiang et al. reveal that AI-resistant skills, network centrality, and proactive personality foster collaboration, while digital identity reconstruction reorganizes participation and authority. Together, these insights portray AI not merely as an instrument of efficiency but as a transformative agent that redefines human creativity and purpose.

Yet as AI permeates every stratum of life, it also exposes humanity to profound ethical and psychological dilemmas. Chen et al. propose governance models with adaptive trust-repair mechanisms—tailoring attribution and social support to failure contexts while using anthropomorphic cues to sustain resilience. Fu et al. call for frameworks that balance technological utility with emotional wellbeing, highlighting the fragility of end-of-life AI applications where algorithms intersect with grief and post-humous identity. Drawing on Foucauldian notions of subjectivation, they warn that AI mourning tools may reconstitute moral agency beyond death itself. Meanwhile, Thomas and Manalil underscore the urgency of algorithmic transparency to mitigate emotional coercion and cognitive dissonance. Their depiction of shadow banning as “digital silence” reveals its erosion of self-perception and autonomy, urging oversight of both visible and subtle algorithmic harms. Collectively, these perspectives affirm that effective governance must weave together trust, ethics, and psychological awareness to ensure that AI systems remain profoundly humane.

Taken together, these insights illuminate a profound reciprocity: humans endow artificial intelligence with creativity, purpose, and moral direction, even as AI amplifies human potential and reshapes the horizons of thought and collaboration. The evolving discourse on roles and identities thus offers forward-looking pathways for understanding how humanity constructs, safeguards, and enacts subjectivity within an increasingly algorithmic world. As intelligent systems weave themselves ever more deeply into the fabric of life, the imperative becomes clear—to ensure that innovation never eclipses emotion, conscience, and dignity (Bankins and Formosa, 2023). These reflections chart a transformative journey toward self-realization in the digital epoch and toward governance structures capable of reconciling technological power with ethical responsibility.

Ultimately, this corpus of scholarship converges upon the global aspiration for “AI for social good.” It reminds us that the true horizon of progress does not reside in the perfection of machines, but in the deepening of our humanity—the enduring capacity to endow intelligence, whether human or artificial, with compassion, justice, and dignity.
